# Multiphase Flow Regime Characterization and Liquid Flow Measurement Using Low-Field Magnetic Resonance Imaging

**DOI:** 10.3390/molecules26113349

**Published:** 2021-06-02

**Authors:** Rutger R. Tromp, Lucas M. C. Cerioni

**Affiliations:** 1 KROHNE New Technologies, Kerkeplaat 12, 3313 LJ Dordrecht, The Netherlands; r.tromp@krohne.com; 2 Department of Applied Physics, Eindhoven University of Technology, Den Dolech 2, 5600 MB Eindhoven, The Netherlands

**Keywords:** low-field magnetic resonance, imaging, multiphase, flow measurement, pipe flow, two-phase flow, flow regime characterization, intermittent flow, slug flow, process and reaction monitoring

## Abstract

Multiphase flow metering with operationally robust, low-cost real-time systems that provide accuracy across a broad range of produced volumes and fluid properties, is a requirement across a range of process industries, particularly those concerning petroleum. Especially the wide variety of multiphase flow profiles that can be encountered in the field provides challenges in terms of metering accuracy. Recently, low-field magnetic resonance (MR) measurement technology has been introduced as a feasible solution for the petroleum industry. In this work, we study two phase air-water horizontal flows using MR technology. We show that low-field MR technology applied to multiphase flow has the capability to measure the instantaneous liquid holdup and liquid flow velocity using a constant gradient low flip angle CPMG (LFA-CPMG) pulse sequence. LFA-CPMG allows representative sampling of the correlations between liquid holdup and liquid flow velocity, which allows multiphase flow profiles to be characterized. Flow measurements based on this method allow liquid flow rate determination with an accuracy that is independent of the multiphase flow profile observed in horizontal pipe flow for a wide dynamic range in terms of the average gas and liquid flow rates.

## 1. Introduction

Within the petroleum industry there is a long-standing need for operationally robust, low cost, and real-time wellhead metering systems with accuracy across a broad range of produced volumes and hydrocarbon properties. Installations currently rely predominantly on accurate, yet costly, and operationally cumbersome test separators that by design deliver time-averaged multiphase flow rate data in well tests spanning several hours, thereby losing real-time flow information [1]. The real-time alternative to multiphase test separators, multiphase flow meter technology, has considerably improved in accuracy over the last decades. However, due to the complex combination of measurement technologies within these systems, these devises are highly sensitive to hydrocarbon properties and require repeated calibration in the field [2,3]. In addition, these multiphase flow meter systems tend to have a limited dynamic range in terms of produced volumes and associated multiphase flow profiles [1,2,3]. This poses problems in field applications as flow regimes can change over time due to natural production transients that can occur over the scale of hours or days, and inescapably occur over the lifetime of a well or due to flow restrictions caused by pipeline fouling that builds up over time during production [4]. In the absence of simple and accurate, plug-and-play well head metering solutions, many wells are operated with insufficient metering leading to suboptimal reservoir management and uncertainty in production allocation to individual wells [1,2,3].

For many years, magnetic resonance-based downhole logging tools have been successfully applied to in situ Earth formation evaluation [5]. These tools apply low field, time-domain magnetic resonance (MR) technology under challenging environmental conditions, proving the robustness of the technology. In the laboratory, the same technology can be used to obtain production fluid composition information from samples [6,7]. In the last decade, considerable effort has been spent to merge the two aspects and apply MR technologies in the process industry in pursuit of industry 4.0 compatible inline process monitoring and control [8]. Real-time wellhead metering systems are a concrete example of such inline process monitoring systems. Several MR technology-based research instruments have been developed for the petroleum industry, showing specific advantages of low-field MR technology when applied to multiphase flow measurement [1,9,10,11,12]. In this article, we show that a fully integrated multiphase flow meter [13] using low-field MR technology can act as a smart and robust measurement platform that has a large dynamic range in terms of produced volumes and associated multiphase flow profiles. This multiphase flow measurement platform can be applied as a general monitoring instrument in chemical and process control industries [8].

The complex flow profiles observed in multiphase flow emerge due to the differences in densities and viscosities of the fluid phases present in the flow [2]. For the case of two-phase, gas-liquid flows, these differences are maximum and the most challenging flow profiles occur. In this article, we focus on the horizontal pipe flow of water and air at atmospheric pressure. These fluids provide several advantages: Firstly, they are chemically safe, simplifying a flow loop design and operation; secondly, at atmospheric pressure the largest difference in gas and liquid density is obtained, leading to the most challenging flow profiles; and thirdly, water-based flows are relevant to a wide variety of processes in the chemical industry, including high water-fraction oil production in the petroleum industry.

**Figure 1:**
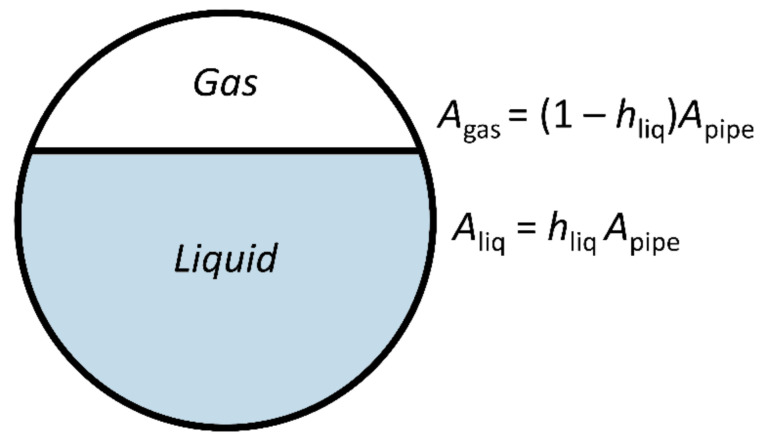
Schematic representation of the definition of the liquid holdup *h*_liq_.

In a static situation, there is a gravity induced separation between the two phases in a gas-liquid mixture present in a horizontal pipe: Gas is concentrated at the top of the pipe and liquid is concentrated at the bottom of the pipe, see Figure 1. When a pressure gradient is added along the length of the pipe, flow is induced. Since the phases have different densities and viscosities, the flow velocity associated with a given pressure drop per unit length is different for each phase. This so-called phase slip between phases is the primary complication in two-phase flows as it creates a dynamic pressure between the two phases. Depending on the cross-sectional area occupied by the two phases, which are commonly expressed using the dimensionless liquid holdup *h*_liq,_ see Figure 1, such that the cross-sectional area occupied by liquid is given by

(1) $$A_{\mathrm{liq}}=h_{\mathrm{liq}}A_{\mathrm{pipe}},$$

where 0 ≤ *h*_liq_ ≤ 1, and *A*_pipe_ is the pipe cross-sectional area, the surface tension of the fluid interface may or may not be strong enough to keep a stable interface between the two phases at a certain phase slip. If it is not, an instability in the local liquid holdup is induced. These instabilities can take the form of small, symmetric waves on a relatively stable fluid interphase, referred to as stratified flow; can give rise to large and chaotic wave patterns reminiscent of rough seas, referred to as wavy flow; and can even lead to such large instabilities that liquid is sucked up to the top of the pipe, creating so-called liquid slugs that are pushed along by the gas at high velocities.

**Figure 2:**
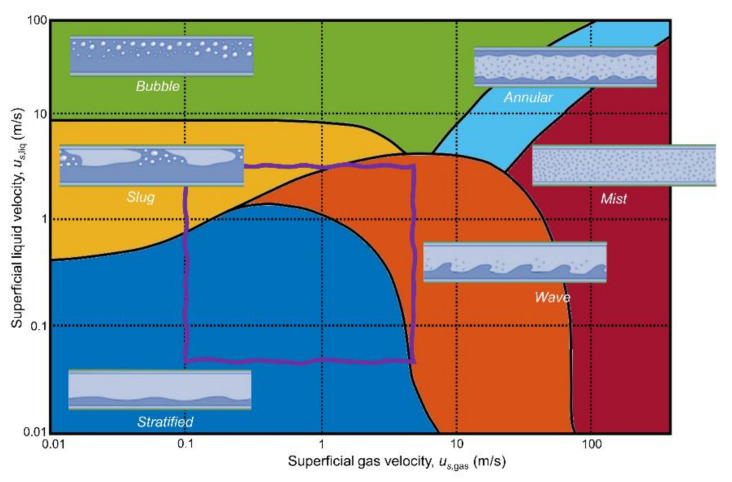
Schematic representation of a two-phase, gas-liquid flow map indicating the multiphase flow patterns likely to occur for a given combination of superficial flow velocities, adapted from [2]. The purple rectangle indicates the superficial gas and liquid flow velocities that can be obtained using the multiphase flow loop used in this study.

Which flow pattern occurs in a given situation depends on many factors of which the upstream and downstream piping configuration are of paramount importance. For a given piping configuration and given volumetric liquid and gas flow rates, the flow pattern can be roughly estimated based on the superficial gas flow velocity, *u*_s,gas_ = Q_gas_/*A*_pipe_, and the superficial liquid flow velocity, *u*_s,liq_ = Q_liq_/*A*_pipe_, where Q*_i_* is the volumetric flow rate of phase *i*. The superficial flow velocity thus represents the fictitious flow velocity of a single phase of a multiphase flow that it would have if all other phases in the multiphase flow were absent from the flow. Figure 2 shows an example flow map for two-phase, gas-liquid flow in a horizontal pipe section that uses the concept of the fictitious superficial flow velocity for parametrization [2]. The purple rectangle in Figure 2 indicates the superficial gas and liquid flow velocities that can be obtained using the multiphase flow loop used in this study. Details about this flow loop are presented in Section 4. Based on this flow map, we may expect stratified, wavy, and slug flow to be observed during multiphase flow experiments.

Based on the preceding discussion of multiphase flow patterns, one can see that correlations between instantaneous holdup and instantaneous flow velocity need to be characterized to accurately determine the flow rates of the individual phases in a two-phase flow.

**Figure 3:**
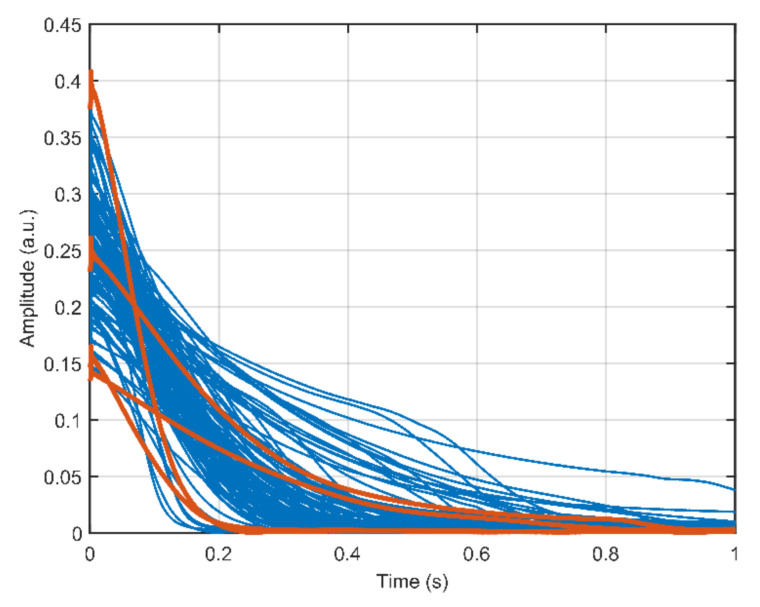
CPMG signal amplitude as a function of time shown for a set of measurements performed on a slug flow-like water-air multiphase flow. The two pairs of CPMG signals printed in red show that different signal amplitudes at *t* = 0 s can occur for the same signal amplitude decay time.

An intuitive and simple method to measure the instantaneous liquid holdup would be to use the MR signal amplitude. However, for samples flowing through an industrial MR system this signal amplitude may depend on other factors than the liquid holdup alone. For instance, consider the CPMG pulse sequence [14,15] measurements presented in Figure 3 for a water-air slug flow-like flow regime. Both the amplitude at *t* = 0 s and the signal amplitude decay time of the CPMG signals vary considerably between measurements. Since air does not contribute to the CPMG signal in this experiment and water relaxation (*T*_2_ ~ 2–3 s) is slow compared to the signal decay time, the signal decay time correlates with flow velocity *u*_liq_ [9]. The signal amplitude correlates strongly with *h*_liq_, although the spin residence time in the polarizing magnetic field influences the observed signal amplitude as well, complicating the direct conversion of signal amplitudes to liquid holdups. The four CPMG signals highlighted in red in Figure 3 show that a given signal decay time or liquid flow velocity, can be observed for multiple signal amplitudes or liquid holdups. The major complication in multiphase flow measurement consequently is that the instantaneous flow rate, i.e.,

(2) $$Q_{\mathrm{liq}}\left(t\right)=u_{\mathrm{liq}}\left(t\right)h_{\mathrm{liq}}\left(t\right)A_{\mathrm{pipe}},$$

needs to be sampled in a way that ensures representative sampling of all characteristic flow events. When representative sampling is achieved, the average liquid flow rate <Q_liq_> during a given time interval is given by the mean of the discrete set of flow events sampled during that time interval, i.e.,

(3) $$Q_{\mathrm{liq}}=u_{\mathrm{liq}}\left(t\right)h_{\mathrm{liq}}\left(t\right)A_{\mathrm{pipe}}≅u_{\mathrm{liq},i}h_{\mathrm{liq},i}A_{\mathrm{pipe}}.$$

MR imaging (MRI) techniques may provide a direct measurement of liquid holdup, which is, for example, independent of the magnetic history of the sample. When MRI sequences are implemented, a spatially varying magnetic field or gradient **G**, is introduced in addition to the main magnetic field $B_{0}$. The effect of introducing the gradient **G** is that the resonance frequency of the nuclear spins varies with the position. The resonance frequency can thus be used to encode the position of nuclear spins. When MRI is applied in presence of flow, translational motion information can be extracted combining an imaging sequence with a spatially resolved measurement of molecular displacement. In many flow MRI studies, the velocity of a fluid media is measured by time-of-flight (TOF) [16,17,18] and phase shift methods [19]. A comprehensive review of non-medical flow MRI methods can be found in the articles by Gladden and Sederman [19,20]. The principles and relevant theory of flow MRI can be found in the books by Callaghan [21,22]. The fundamental concepts of MRI are discussed in an intuitive manner by McRobbie et al. [23].

Here, we focus on obtaining the bulk liquid flow velocity *u*_liq_ from the convective amplitude decay of the CPMG signals that is induced by the outflow of the excited sample volume [9]. We combine the CPMG pulse sequence with an external gradient *G*_z_ applied in the transversal vertical direction of the pipe to obtain a one-dimensional spatial distribution of the liquid, which we refer to as an one-dimensional (1D) distribution image. There are several techniques that combine the CPMG pulse sequence with an imaging sequence for spatial encoding [24]. As typical pulse sequences based on phase encoding gradients may increase the total acquisition time [24], we use frequency encoding to spatially encode all the points simultaneously during one CPMG spin-echo train. Since pulsed or modulated gradients require highly complex power electronics and gradient coils design that in an industrial application are translated into complexity for manufacturing, we use a constant-gradient CPMG [25]. This implementation additionally provides the advantage of short echo spacing for the convective amplitude decay velocity measurement. The frequency encoded spatially resolved 1D distribution image, can be obtained from the Fourier transform of each individual spin-echo signal [25].

To maximize the resolution and minimize the blurring effect due to inhomogeneities by spatial variations in $B_{0}$ [26], we want to apply the maximum gradient strength available. During the application of the constant gradient *G*_z_, the spectral width of the RF-pulses, Δν_RF_, must be larger or equal to the spectral width of the sample, Δν_sample_. The spectral width of the sample is given by Δν_sample_ = γ*G*_z_*D*/2π, where *γ* is the gyromagnetic ratio of the proton, and *D* is the pipe diameter [25]. The spectral width of a rectangular RF pulse can be approximated by Δν_RF_ ≈ 1/*t*_pulse_, where *t*_pulse_ is the RF pulse duration. The pulse sequence design relation between RF pulse length and applied gradient strength may thus be written as

(4) $$t_{\mathrm{pulse}}\leq \frac{2\pi }{\gamma G_{z}D}.$$

When we apply the maximum gradient strength *G*_z_ in our application, both 90° excitation and 180° refocusing pulses as used in a standard CPMG pulse sequence do not fulfill Equation (4). In other words, a standard CPMG would have limited bandwidth and cannot be used to excite and monitor the convective amplitude decay over the full pipe cross-section. This limitation was overcome by using a low flip angle CPMG (LFA-CPMG) [27], where all RF pulses are substituted by short duration pulses. This way the LFA-CPMG pulse sequence allows the instantaneous liquid holdup *h*_liq_ to be derived from the 1D liquid distribution image obtained from frequency encoded spin-echo signals, while the instant liquid flow velocity *u*_liq_ can be determined from the effective convective amplitude decay of the LFA-CPMG signals.

In this article, we will show that low-field MR technology applied to multiphase flow has the capability to measure the instantaneous liquid holdup and liquid flow velocity using the constant gradient LFA-CPMG pulse sequence. To this end, we applied the LFA-CPMG to study two-phase air-water flow experiments. The details of the experimental method and setup are presented in Section 4. In the following section it will be shown that LFA-CPMG allows the correlations between liquid holdup and liquid flow velocity to be determined, and it is shown that flow profiles can be identified based on these correlations. In addition, we show that flow calculations based on these correlations allow liquid flow rate determination with an accuracy that is independent of the multiphase flow profile observed in a horizontal pipe flow for a wide dynamic range in terms of the average gas and liquid flow rates.

## 2. Results

**Figure 4:**
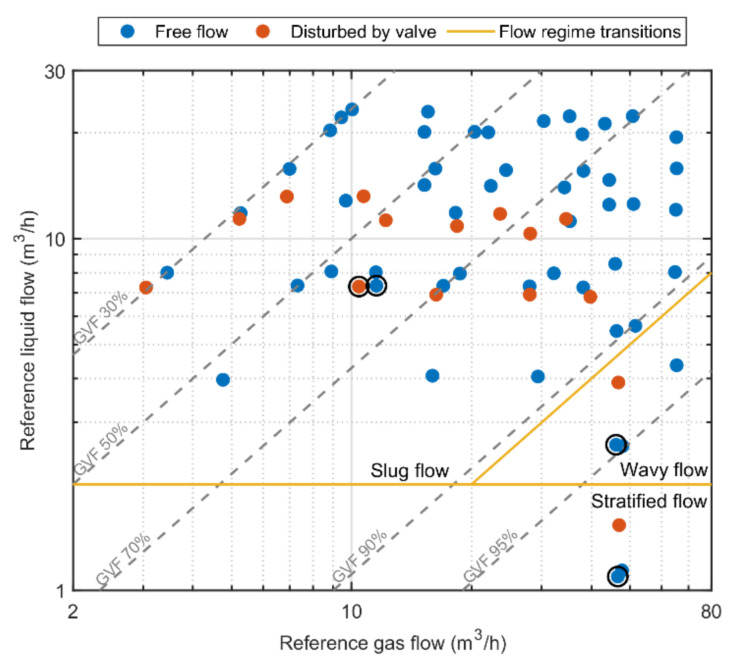
The measured single phase reference flow rates for all two-phase air-water flow experiments performed in this study indicated as dots. The dashed lines indicate the gas volume fraction (GVF) of the multiphase flows. Flow experiments have been performed for free flow and for flow disturbed by a downstream valve. Approximate flow regime transition boundaries were derived from visual inspection and are indicated by the solid lines. Four experiments that are discussed in more detail in this article are highlighted by a black circle.

The set of two-phase air-water flow experiments that were performed is shown in Figure 4. Flow experiments have been performed for free flow and for flow disturbed by a downstream valve. This downstream ball valve closes in the vertical direction and was for 25% opened in the disturbed flow experiments. Flow regimes were identified for each flow experiment based on the multiphase flow profiles observed through a transparent pipe section. Stratified, wavy, and slug flow regimes were observed during the flow experiments and snapshots of typical gas and liquid phase distributions in these flows are indicated in Figure 2. Based on these visual identifications flow regime transition boundaries could be identified and these are indicated by the solid lines in Figure 4. Dashed lines indicate the gas volume fraction (GVF) of the multiphase flows, i.e., GVF = Q_gas_/(Q_gas_ + Q_liq_). Four experiments are highlighted by a black circle. These experiments are discussed in more detail in this article as examples. Video footage is made available in the supplementary information for some example experiments to illustrate dynamic liquid holdup variations occurring in gas-liquid multiphase flow.

In each flow, experiment data were acquired for 30 min using the low-field MR technology-based multiphase flow measurement method that is discussed in detail in Section 4. This measurement method uses a broadband excitation, constant-gradient LFA-CPMG pulse sequence to derive liquid holdup information from 1D liquid distribution images obtained from frequency encoded spin-echo signals, while liquid flow velocity information is derived from the convective amplitude decay of the LFA-CPMG signals with time as induced by the outflow of the excited sample volume. The frequency distribution of each spin-echo that is induced by the gradient field along the vertical direction represents a distribution image along the height of the pipe of the liquid portion of the flow, as air does not give an MR signal. This imaging functionality can be used to determine the multiphase flow profile in a given flow experiment from the combined liquid distribution images acquired during the 30 min of data acquisition.

### 2.1. Liquid Distribution Image Interpretation

**Figure 5:**
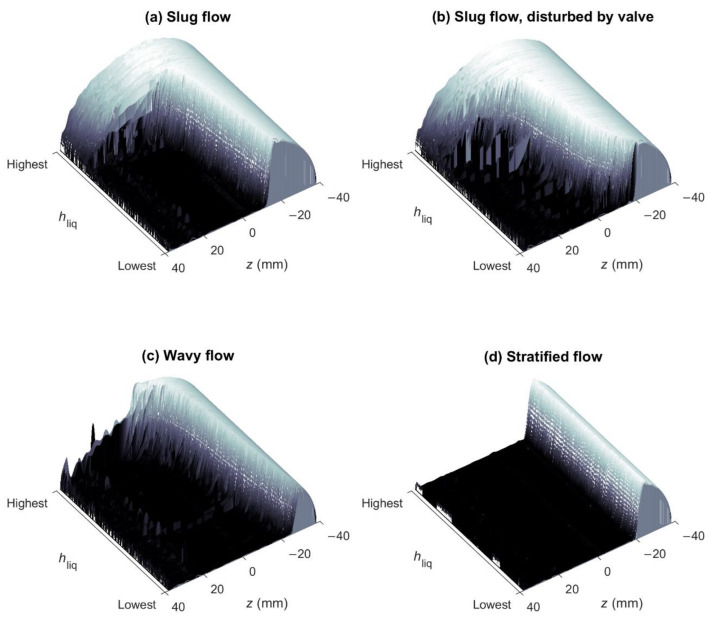
The set of liquid distribution images acquired in four different flow experiments represented as a surface plot in which liquid distribution images are sorted from the highest to lowest measured holdup. The axis labeled as *z* indicates the height along the flow tube with the pipe axis located at *z* = 0 mm. Each experiment corresponds to a unique multiphase flow profile: (**a**) Slug flow (Q_gas_ = 11.5 m^3^/h and Q_liq_ = 7.3 m^3^/h); (**b**) slug flow, disturbed by valve (Q_gas_ = 10.4 m^3^/h and Q_liq_ = 7.3 m^3^/h); (**c**) wavy flow (Q_gas_ = 46.1 m^3^/h and Q_liq_ = 2.6 m^3^/h); and (**d**) stratified flow (Q_gas_ = 46.6 m^3^/h and Q_liq_ = 1.1 m^3^/h).

Figure 5 shows the set of liquid distribution images acquired for the four experiments that are marked by a black circle in Figure 4. A surface representation of the liquid distribution images is used in which the images are sorted from the highest to lowest measured holdup to create a smooth surface that is more easily compared between experiments. For a full pipe of water, the liquid distribution image would take on the form of a semicircle and these conditions occur for about 25% of the time in the slug flow experiment (Q_gas_ = 11.5 m^3^/h and Q_liq_ = 7.3 m^3^/h) shown in Figure 5a. The remainder of the time corresponds to a steady flow situation in which the pipe is partially filled with a constant liquid fraction. Slug flow can thus be envisioned as a binary flow system with two main events: Short bursts of liquid slugs with *h*_liq_ ≈ 1, and longer events in which gas is accumulated at the top of the pipe and liquid at the bottom. This latter phase is very much comparable to the stratified flow experiment (Q_gas_ = 46.6 m^3^/h and Q_liq_ = 1.1 m^3^/h) presented in Figure 5d and is often referred to as the film phase of the slug flow.

The slug flow experiment was repeated with the flow disturbed by the downstream valve (Q_gas_ = 10.4 m^3^/h and Q_liq_ = 7.3 m^3^/h), in order to induce a more unstable flow profile. Figure 5b shows that although the flow profile can in general still be classified as slug flow, the valve disturbance leads to a considerably altered liquid distribution image surface, especially in the film phase. Although still about 15% of the time slugs with *h*_liq_ ≈ 1 are observed, there is no longer a steady flow situation in the film phase. The film phase consequently has a liquid fraction in the pipe that changes continuously in time. This situation can be compared to the flow experiment labeled as wavy flow (Q_gas_ = 46.1 m^3^/h and Q_liq_ = 2.6 m^3^/h) that is presented in Figure 5c. The closing of the downstream valve in this flow experiment thus reduced the fraction of liquid slugs and induced wavy flow in the film phase of the slug flow.

### 2.2. Liquid Holdup and Velocity Correlations

**Figure 6:**
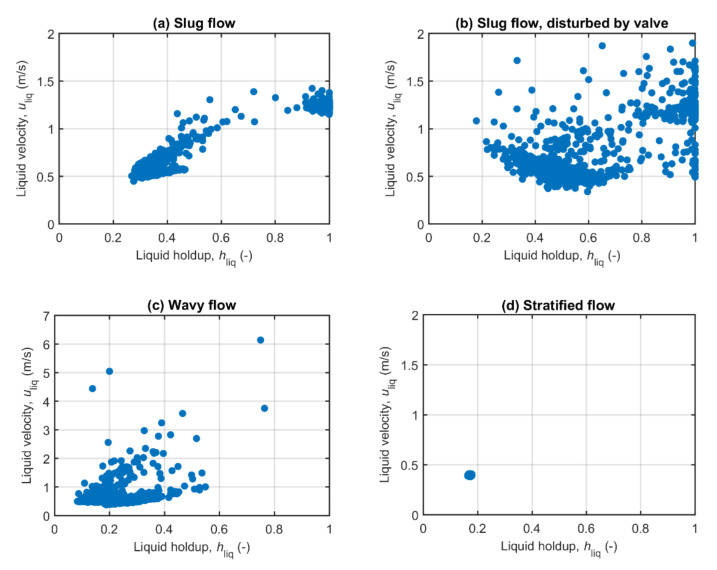
Measured liquid flow velocity as a function of measured liquid holdup for the same four flow experiments as for which the liquid distribution images were presented in Figure 5. These experiments correspond to: (**a**) Slug flow (Q_gas_ = 11.5 m^3^/h and Q_liq_ = 7.3 m^3^/h); (**b**) slug flow, disturbed by valve (Q_gas_ = 10.4 m^3^/h and Q_liq_ = 7.3 m^3^/h); (**c**) wavy flow (Q_gas_ = 46.1 m^3^/h and Q_liq_ = 2.6 m^3^/h); and (**d**) stratified flow (Q_gas_ = 46.6 m^3^/h and Q_liq_ = 1.1 m^3^/h).

As mentioned in the introduction, the accurate calculation of the liquid flow rate in multiphase flow comes down to the task of acquiring the correlations between the instantaneous liquid holdup and liquid flow velocity that are characteristic for a given flow profile. Figure 6 shows these correlations as derived from our low-field MR-based flow measurements for the same four flow experiments as for which the liquid distribution images were presented in Figure 5.

Starting with the simplest case, stratified flow as presented in Figure 6d, a single point correlation is observed. This means that a given liquid holdup is directly related to a given liquid flow velocity. In such cases, the sampling rate and measurement time of the flow measurement method has little influence on the measurement results, as a single measurement already represents a representative sample of the multiphase flow. More structure is visible in the correlation plot for slug flow shown in Figure 6a. The binary character of slug flow is clearly represented by the two main concentrations of data points around *h*_liq_ = 0.3 (film phase) and around *h*_liq_ = 1 (slug phase). Note the higher flow velocity of about 2.5 times in the slug phase of the flow. Recalling that about 25% of the time the flow can be associated with the slug phase, most of the liquid flow is transported by the slug phase. This shows the importance of representative sampling of the flow, as even the minor under sampling of the slug phase may lead to large flow measurement errors.

The disturbed slug flow (Figure 6b) and wavy flow (Figure 6c) experiments exhibit a large spread in the flow velocities that are observed at a given liquid holdup. This spread consequently signals that complex stochastic processes are describing the correlations between the instantaneous liquid holdup and liquid flow velocity. Sufficiently fast sampling is expected to be very important for the accurate measurement of the liquid flow rate for these seemingly chaotic flow profiles. The fact that even for these flows clusters of data points are clearly observable in Figure 6, provides an indication that the statistics of these flows is sufficiently sampled, thus ensuring a representative sampling set of the liquid holdup and flow velocity correlations in the flow.

### 2.3. Liquid Flow Rate Measurement Accuracy

It is rather straightforward to compute the average liquid flow rate once the instantaneous liquid holdup and liquid flow velocity correlation is available. Assuming a statistically representative sample of the correlation is obtained by taking a total of *N* measurements, the average liquid flow rate may be computed as the average of the point-by-point product of liquid holdup and velocity, multiplied by the area of the pipe, i.e.,

(5) $$\langle Q_{\mathrm{liq}}\rangle =\frac{A_{\mathrm{pipe}}}{N}\overset{N}{\underset{i=1}{\sum }}h_{\mathrm{liq},i}u_{\mathrm{liq},i}.$$

**Figure 7:**
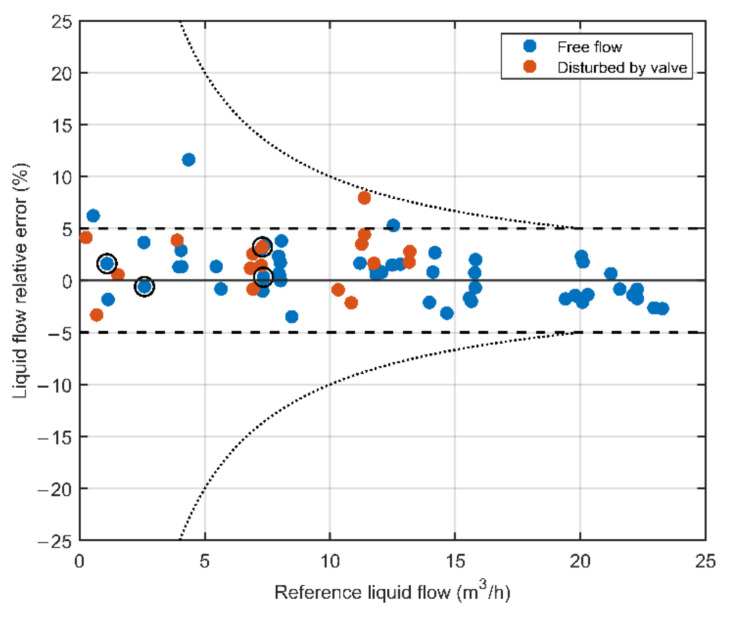
The relative liquid flow rate error as a function of reference liquid flow rate for all multiphase flow experiments that were presented in Figure 4. The dashed lines represent a ±5% error band that is the generally accepted liquid flow rate accuracy required in multiphase flow metering. The dotted lines represent the relative error that corresponds to a zero-point inaccuracy of ±1 m^3^/h that is commonly accepted as a practical limit for the accuracy of multiphase flow metering systems at low liquid flow rates.

Figure 7 shows the relative liquid flow rate error as a function of the reference liquid flow rate for all experiments presented in Figure 4 together with an ±5% error band that is the generally accepted liquid flow rate accuracy required in multiphase flow metering (dashed lines) [12] and the relative error that corresponds to a zero-point inaccuracy of ±1 m^3^/h (dotted lines) that is commonly accepted as a practical limit for the accuracy of multiphase flow metering systems at low liquid flow rates [2]. The relative flow error is within the ±5% error band for all but four flow experiments. No difference in flow accuracy is observed between the flow experiments that had free flow or were disturbed by the downstream valve.

## 3. Discussion

The multiphase flow profile independent liquid flow rate measurement accuracy presented in Section 2 is remarkable, considering the highly complex liquid holdup and liquid flow velocity correlations that are observed in these multiphase flows (see Figure 6), especially when the flow was disturbed by the downstream valve. Therefore, these results prove that a low-field MR-based flow metering apparatus can be applied to ensure representative sampling of the multiphase flow in a wide range of multiphase flow profiles and a wide dynamic range in terms of the average gas and liquid flow rates. In addition, this proves that flow regime identification is possible using MR measurement methods, which can be an important asset in the industry. For instance, in oil production and process optimization, where slugs may induce excessive structural vibration in piping systems causing component failures due to fatigue or resonance [2,28,29]. Research into the multiphase flow can benefit from measurement equipment that does not disturb the flow. Finally, the frequency encoding-based liquid holdup determination method applied in this study is shown to be robust enough to be applied to multiphase flows, opening up MR-based imaging opportunities in industrial multiphase flow applications.

## 4. Materials and Methods

**Figure 8:**
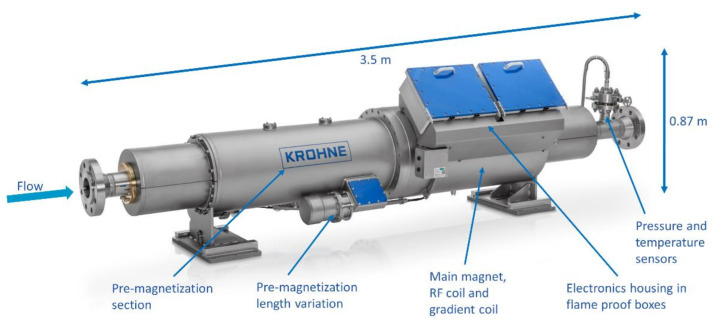
Annotated photograph of the KROHNE MPHASE 5000 MR-based multiphase flow meter used for the flow experiments presented in this work. Image courtesy of KROHNE [30].

The flow experiments presented in this work were performed using the M-PHASE 5000 multiphase flow meter developed by KROHNE [30] and shown in Figure 8. The 3.5 m long instrument is designed around a horizontal glass fiber reinforced epoxy (GRE) flow tube that is available in 2″, 3″, and 4″ pipe sizes. A 3″ pipe was used in this work, which has an 80 mm internal diameter. The main magnet section was constructed using a two-ring, 90 cm long, 0.2 T Halbach magnet with a length-to-radius ratio of 6. It contains a cylindrical region-of-interest (ROI) of 10 cm length and 10 cm diameter that was passively shimmed to a homogeneity of about 1000 ppm. A 12.5 cm long solenoid-shaped volume coil with an inner diameter of 12 cm, and a 40 cm long *z*-gradient coil with an inner diameter of 15 cm are centered on the ROI. The RF coil was used for both transmission of RF pulses and reception of NMR signals and was driven at 8.5 MHz using an RF power of 1.3 kW. The gradient coil was operated using a continuous current that generated a gradient field strength of 23.5 mT/m (equivalent to 10 kHz/cm). All electronics required for the NMR measurements and data transfer to a control computer are integrated into two flame-proof boxes that are mounted directly onto the flow meter. The instrument is additionally equipped with a pre-magnetization section consisting of 3 identical, two-ring, 30 cm long, unshimmed, 0.2 T Halbach magnets. The pre-magnetization length can be varied by selectively activating pre-magnetization sections by rotating the inner ring with respect to the outer ring in the Halbach section by 180 degrees.

**Figure 9:**
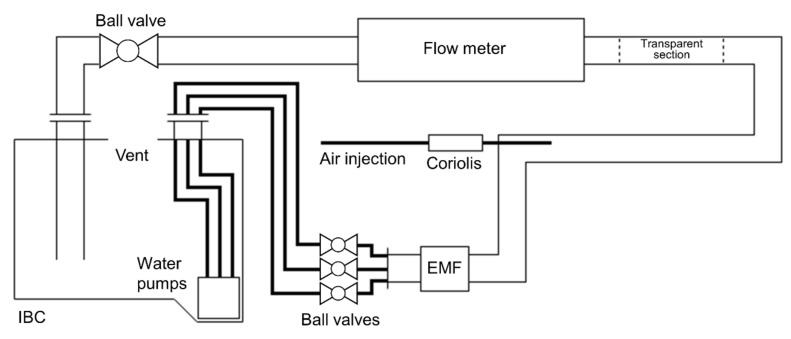
Schematic representation of the two-phase water-air flow loop used for the flow experiments presented in this work. Note that the air injection point, flow meter, and downstream ball valve of the flow loop were all placed at the same elevation above the IBC, making the piping horizontal over the entire length of the two-phase flow path.

The flow experiments presented in this work were performed on water-air mixtures for a wide range of flow rate combinations using the maximum pre-magnetization length. A schematic representation of the flow loop used is presented in Figure 9. Water flow was controlled by using 3 commercially available submersible garden water pumps that could be powered on independently. These pumps were placed in a 1 m^3^ Industrial Bulk Container (IBC) tank and yielded a combined maximum water flow rate of 48 m^3^/h. The flow loop was kept at atmospheric pressure via a vent in the IBC. The water injection point in the flow tubing for each pump was fitted with a ball valve that allowed for fine-tuning of the water injection for each individual pump. Whenever a pump was inactive, the ball valve allowed this pump to act as a controlled bypass for lowering flow rates through the magnet. This way, the superficial water flow velocity could be varied from about 0.5 cm/s up to 3 m/s. Air injection from a central laboratory compressed air supply was controlled using a needle valve and the superficial gas flow velocity could be varied from about 5 cm/s up to 3.5 m/s, corresponding to a maximum gas flow rate of 60 m^3^/h through the multiphase flow meter. The reference volumetric flow rate for injected water was measured using a commercial electromagnetic flow meter (EMF in Figure 9) that has an accuracy better than 0.2% [31], while a commercial Coriolis flow meter with accuracy better than 0.5% [32] was used for air mass flow measurement. The air mass flow rate was converted to a volumetric air flow rate using dry air PVT calculations [33] based on the temperature and pressure measurements that are integrated into the M-PHASE 5000 multiphase flow meter. The flow loop piping layout was U-shaped and had a total straight flow length of 2 m (25D) applied upstream and downstream of the multiphase flow meter for flow conditioning. A ball valve was added just before the flow return connection to the IBC tank, which allowed the effect of flow disturbances on multiphase flow profiles and multiphase flow measurement accuracy to be studied by partially closing this valve. Multiphase flow profiles during the tests could be observed through the 1.5 m long transparent pipe section placed in front of the multiphase flow meter. Some example flow profile videos captures are provided in the supplementary information. Based on the observations during the tests, a flow map could be created to help predict the flow profile in the flow loop as a function of the gas and liquid flow rates. This flow map was presented as Figure 4 in Section 2.

**Figure 10:**
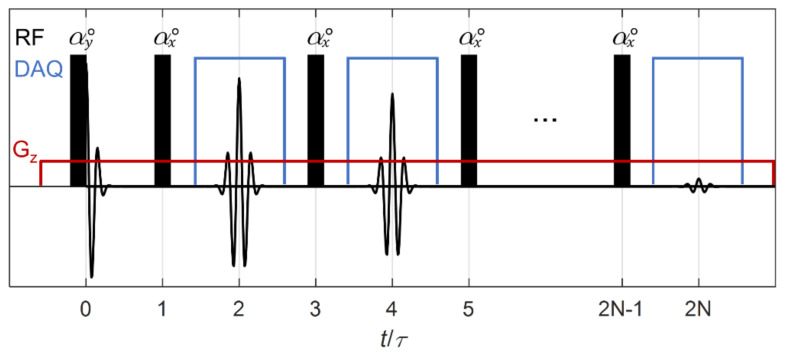
Schematic representation of the broadband excitation constant-gradient LFA-CPMG pulse sequence used in flow experiments. RF pulses with on-resonance flip angle α° are indicated by black rectangles. Digital acquisition (DAQ) of spin-echoes is represented in blue. The field gradient *G*_z_ is represented in dark red.

Flow experiments were performed using broadband excitation constant-gradient LFA-CPMG pulse sequences [33,34] using 45° flip angle pulses of duration *t*_pulse_ = 10 µs, and 2*τ* = 800 μs echo spacing. This pulse sequence is shown schematically in Figure 10. To ensure the maximum initial signal amplitude and uniform spectral width of both excitation and refocusing pulses, pulse duration was kept the same for both excitation and refocusing pulses. Low flip angle pulse sequences can be used to determine the frequency spectrum of the sample in the ROI even in situations where limited SNR is available by combining the data from several echoes [35]. In addition, the amplitude decay of the LFA-CPMG signals with time due to the convective outflow of spins from the ROI, as obtained from the envelope of the spin-echo maximum amplitudes, can be used to derive average flow velocity information [9]. The number of acquired echoes and the wait time between consecutive pulse sequence executions were optimized in each flow experiment using the integrated flow measurement optimization feature of the KROHNE M-PHASE 5000. This algorithm actively tunes the number of echoes in real-time to match the lowest flow velocity component that occurs in the multiphase flow during the flow experiment. The wait time is set to 2 times the echo train length to ensure the sample is fully refreshed between consecutive pulse sequence executions. The liquid holdup was obtained by integrating the liquid distribution image obtained from the first 20 echoes and taking the ratio of this integral with the integral of a full pipe water reference measurement.

Prior to the two-phase flow experiments, velocity determination was calibrated, and liquid distribution image-based liquid holdup determination was validated.

**Figure 11:**
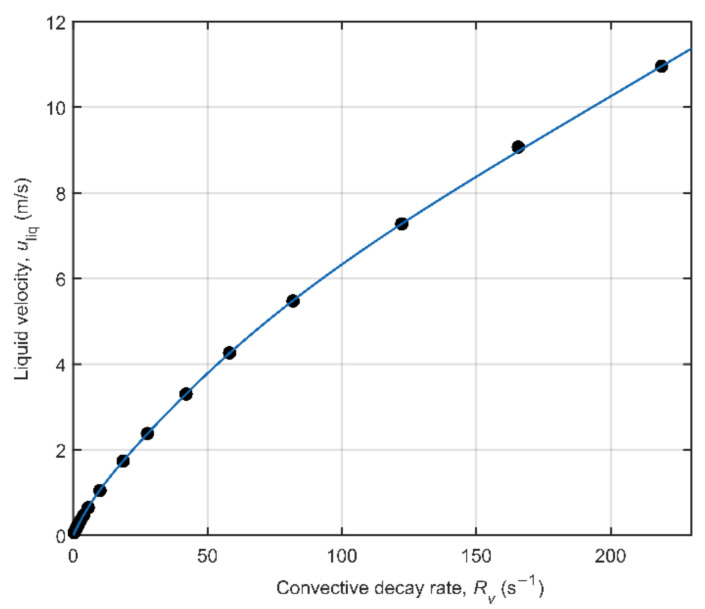
The reference liquid flow velocity, *u*_liq_, as a function of the convective decay rate, *R_v_*, that was obtained by fitting an exponential decay to *T*_2,eff_-corrected LFA-CPMG signals. The solid line indicates the calibration function used in the two-phase flow experiments.

Pure water flow experiments were performed on a dedicated single phase flow loop at KROHNE to calibrate the slope of the flow velocity determination via the convective decay of the LFA-CPMG signals. Figure 11 shows the relation between the reference flow velocity and the convective decay rate, *R_v_*, for 17 different flow velocities up to 11 m/s. This convective decay rate was determined by fitting an exponential decay to *T*_2,eff_-corrected LFA-CPMG signals. The *T*_2,eff_ used for correction was determined as the effective *T*_2_ decay obtained from a static LFA-CPMG experiment performed prior to each flow experiment. The use of an exponential convective decay model is based on the work by Petrova et al. [34] that showed the asymptotic form of the *T*_2,eff_-corrected signal in low flip angle CPMGs to be exponential. The exponential fit was validated to be a better fit to our data than the linear fit method that is applied in flow measurement using bulk CPMGs.

**Figure 12:**
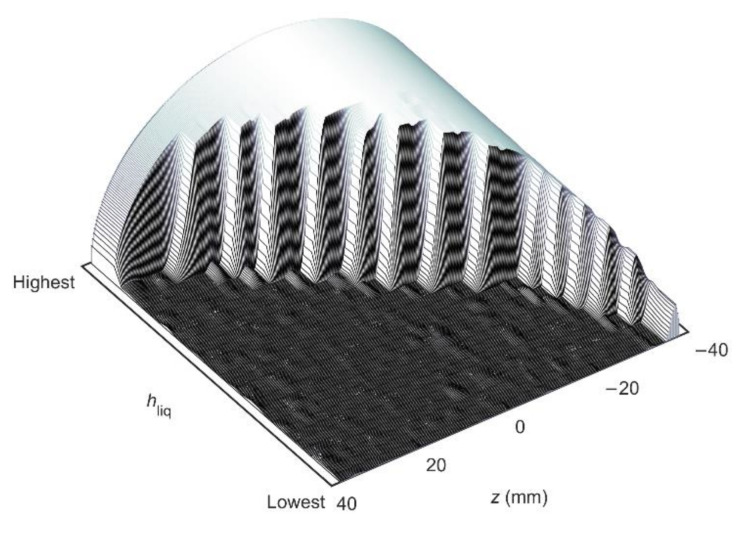
The set of 1D liquid distribution images acquired in the static verification experiments represented as a surface plot in which liquid distribution images are sorted from the highest to lowest measured holdup. The axis labeled as *z* indicates the height along the flow tube with the pipe axis located at *z* = 0 mm. The separate experiments are well recognized as steps in the surface as a progressively bigger portion of the semi-circle is cut-off due to the absence of liquid.

**Figure 13:**
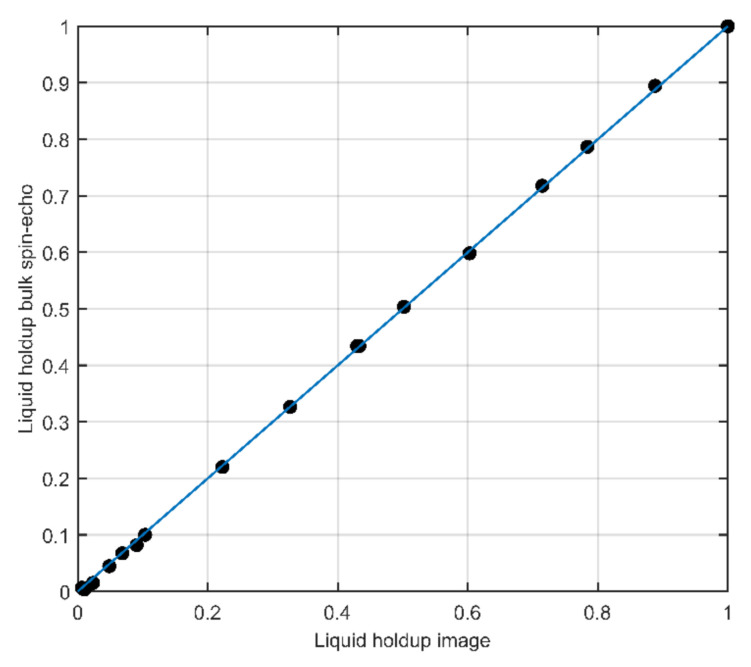
The liquid holdup as determined from a bulk spin-echo experiment as a function of the liquid holdup as determined from the 1D liquid distribution images obtained using a broad band constant-gradient LFA-CPMG pulse sequence. The measurements for 19 different liquid holdups show a 1-to-1 correspondence between the two methods.

The liquid holdup determination was validated by filling the multiphase flow loop shown in Figure 9 completely with water and by draining the piping in a controlled way via the ball valve that is placed downstream of the flow meter. A total of 19 different liquid levels were created this way, ranging from 100% down to 0.5% liquid holdup. The combined 1D liquid distribution images of these liquid level steps are shown in Figure 12, where the full semi-circle at the top left indicates the full pipe liquid experiment. When stepping from this 100% liquid holdup experiment down to lower liquid holdups, a progressively bigger portion of the semi-circle is cut-off due to the absence of liquid. As reference liquid holdup, the signal amplitude of a bulk spin-echo (*A*_BSE_) was used. The reference liquid holdup for experiment *i* can be derived from the bulk spin-echo amplitude using the relation

(6) $$h_{\mathrm{liq},i}=\frac{A_{\mathrm{BSE},i}}{A_{\mathrm{BSE},100\%}},$$

in which *A*_BSE,100%_ is the bulk spin-echo amplitude obtained for a full pipe of liquid_._ The relation between the liquid holdup obtained using the bulk spin-echo and obtained from the liquid distribution images as acquired using the LFA-CPMG frequency-encoded spin-echoes is shown in Figure 13. A one-to-one correspondence between both methods exists over the entire range, indicating the robustness of the liquid distribution image-based liquid holdup determination method, even at liquid holdups down to a few percent.

## 5. Patents

Patent pending, provisional application number is DE 10 2021 111 162.5.

## Data Availability

The data presented in this study are available on request from the corresponding author. The data are not publicly available due to KROHNE Messtechnik GmbH proprietary rights.

## Supplementary Materials

The following videos of example flow experiments are available online. Video S1: Slug_slow_Qgas11.5_Qliq7.3.mp4 (Q_gas_ = 11.5 m^3^/h and Q_liq_ = 7.3 m^3^/h), Video S2: Slug_fast_Qgas43.2_Qliq21.2.mp4 (Q_gas_ = 43.2 m^3^/h and Q_liq_ = 21.1 m^3^/h), Video S3: Wavy_Qgas46.1_Qliq2.6.mp4 (Q_gas_ = 46.1 m^3^/h and Q_liq_ = 2.6 m^3^/h), Video S4: Stratified_Qgas46.6_Qliq1.1.mp4 (Q_gas_ = 46.6 m^3^/h and Q_liq_ = 1.1 m^3^/h).

## Sample Availability

Not available.
